# The Throat and Nose in the Ætiology of Tuberculosis

**Published:** 1909-07-31

**Authors:** 


### Laryngology and Rhinology.

### THE THROAT AND NOSE IN THE ÆTIOLOGY OF TUBERCULOSIS.

A glance at the excellent series of articles collected and edited by Dr. T. N. Kelynack* will serve to show that a large amount of consideration is being devoted under its different aspects to the part played by the nose and fauces in allowing the entrance of the bacillus tuberculosis into the body. It was at one time pretty generally accepted as an axiom that pulmonary phthisis is the result of the direct inhalation into the lungs of tubercle bacilli floating in the air. But experiments on the inhalation of dust showed that this simple explanation cannot be accepted in its entirety. Many series of inhalation and feeding experiments have been performed, and it has been proved that tubercle bacilli can enter the body through the mucous membrane of the fauces and alimentary canal without producing any lesion at the site of entry; the theory is now widely held that the infection of tuberculosis generally obtains admission in this way and passes into the lymphatic system, and there is no doubt that the lungs can be infected by this route, though it is not yet proved in what proportion of cases pulmonary phthisis is actually produced by infection through the lymphatics. It appears to be a fact that in childhood the lymphatic glands are ordinarily attacked, whereas in adults the tendency is to infection of the lungs without obvious involvement of the lymphatics. It is likely that the route by which the lung is infected is often by the cervical lymphatics across the pleura to the apex of lung, and in support of this is the frequent presence of pleural adhesions in the neighbourhood of the pulmonary lesion, and the fact that phthisis is often ushered in by an attack of pleurisy. Evidence is accumulating to show that tuberculous infection usually takes place in early life, and that it may remain latent for a prolonged period in the lymphatic system. In cases of pulmonary phthisis in adults it is quite possible that the disease may be due to bacilli which have entered the system in childhood and remained quiescent for many years.

Children are peculiarly exposed to the infection of tuberculosis, both in milk and in dust, the latter on account of their proximity to the floor and their habit of putting everything into their mouths. Waldeyer's ring of lymphoid tissue, comprising the naso-pharyngeal tonsil or adenoids, the faucial tonsils, and the lingual tonsil, provides frequent opportunity for the entry of the bacillus tuberculosis. Much work has been done in investigating the presence of tuberculous lesions and bacilli in adenoids removed from ordinary, apparently non-tuberculous cases; the results have differed very widely, some authors having found as many as ten per cent. of tuberculous lesions, and six per cent. containing tubercle bacilli. In any case there is no doubt that the bacilli can reach the lymphatic system without producing lesions at the site of entry. Tuberculosis of the cervical glands is certainly caused by infection through adenoids and tonsils. Besides this, simple inflammatory enlargement is extremely common in children, and is most frequently the result of septic absorption from the faucial lymphoid tissue. A chain of shotty glands along the posterior border of the sterno-mastoid is almost pathognomonic of adenoids, and an enlarged gland below the angle of the jaw is very commonly associated with an unhealthy tonsil. It is probable that this inflammatory condition lowers the resistance of the gland and makes it more likely to afford a lodgment to the tubercle bacillus.

It has been estimated that a half to a third of the cases of enlarged cervical glands are tuberculous. Peters mentions three types of gland which commonly contain tubercle bacilli: (1) The large gland, soft to the finger, exhibiting either pearly tubercles or caseous degeneration; such contain tubercle bacilli in large numbers. (2) The large, hard, homogeneous gland, which reveals giant cells and relatively few bacilli. (3) Slightly enlarged nodes, which may contain bacilli, but no tuberculous structure. Tonsils and adenoids are the first large collection of lymphoid tissue met by the air and food; while they subtract a certain number of bacteria they cannot be considered to act as an effective barrier, and, when affected by inflammation, they provide every opportunity for septic absorption. It is a matter of everyday clinical experience that enlargement of cervical glands frequently subsides after removal of unhealthy faucial lymphoid masses. At the same time, the presence of adenoids and tonsils results, in children, in a considerable degree of malnutrition which predisposes to infection, and also produces chest deformities, which have always been considered to be associated with the tendency to phthisis. To sum up, it is clear that the presence of unhealthy masses of lymphoid tissue in the naso-pharynx and fauces not only predisposes to phthisis but is the direct cause of the entrance of tubercle bacilli into the lymphatic system, and it is by no means improbable that this is a common mode of infection in pulmonary tuberculosis.

The action of the healthy nose in filtering the inspired air is so well known that it would naturally be expected that, when this action fails, the tendency to phthisis would be increased; and this will still be the case even if the disease be not due to direct inhalation, for in cases of nasal obstruction the bacilli inhaled by the mouth impinge on the fauces and thus enter the system. Indeed, it is hardly possible that pulmonary infection can occur by direct inhalation through the healthy nose, for air which has passed through the nares has been shown to be sterile. The nasal mucus is not actively bactericidal; the bacilli become entangled in the mucus and are partly removed at the nostrils, partly pass backwards over the fauces where they may be absorbed, and some are swallowed, and may later pass through the intestinal wall. The effect of the

* *Tuberculosis in Infancy and Childhood: Its Pathology, Prevention, and Treatment.* By various writers. Baillière, Tindall, and Cox. 1908.

absence of nasal filtration is definitely shown by the tendency to phthisis among patients on whom tracheotomy has been performed; atrophic rhinitis, also, in which nasal filtration is absent, is generally considered to predispose strongly to phthisis. Nasal obstruction is so common that its effect in the causation of phthisis is somewhat difficult to estimate, but many medical officers to sanatoria consider it to be an important factor, and a large experience at a consumption hospital has given grounds for the opinion that nasal obstruction is far more common among consumptives than among the normal population. In this connection Peters examined 43 consumptives and found 31, or 72 per cent. with definite obstruction, and only four with perfectly free nasal breathing.

